# Thioredoxin interacting protein promotes invasion in hepatocellular carcinoma

**DOI:** 10.18632/oncotarget.26402

**Published:** 2018-12-07

**Authors:** Aysim Gunes, Ezgi Bagirsakci, Evin Iscan, Gulcin Cakan-Akdogan, Umut Aykutlu, Serif Senturk, Gunes Ozhan, Esra Erdal, Deniz Nart, Funda Yilmaz Barbet, Nese Atabey

**Affiliations:** ^1^ Izmir Biomedicine and Genome Center, Izmir, 35340 Balcova, Turkey; ^2^ Ege University, Faculty of Medicine, Department of Pathology, Izmir, 35040 Bornova, Turkey

**Keywords:** HCC, oxidative stress, TXNIP, EMT, metastasis

## Abstract

**Background:**

Considerable evidence suggests that oxidative stress plays an essential role in the progression of hepatocellular carcinoma (HCC). While acquired resistance to oxidative stress is the main driver of aggressive cell phenotype, the underlying mechanisms remain unknown. Here, we tested the hypothesis that elevated expression of Thioredoxin-interacting protein (TXNIP) is a main regulator of the aggressive phenotype in HCC.

**Materials and Methods:**

To test this hypothesis, we measured TXNIP expression levels in 11 HCC cell lines by qPCR and western blotting. In addition, 80 pairs of HCC tissues and matched liver tissues of 73 cases, as well as 11 normal liver tissue samples were examined by immunohistochemistry. Besides, TXNIP expression levels were analyzed by Oncomine Platform in seven independent microarray datasets. Finally, the functional role of TXNIP in HCC was investigated *in vitro* and *in vivo* by silencing and overexpression studies.

**Results:**

Our results show that TXNIP expression is significantly increased in HCC compared to non-tumor counterparts (*p* < 0.0001) as well as to normal (*p* < 0.0001) and cirrhotic (*p* < 0.0001) liver tissues. Moreover, stable overexpression of TXNIP in HCC cells (i) significantly increases ROS levels, (ii) induces EMT phenotype, (iii) increases motility, invasion and 3D branching tubulogenesis, (iv) decreases apoptosis, and (v) elevates *in vivo* metastasis in zebrafish embryos. Finally, we identify sinusoidal/stromal and cytoplasmic TXNIP staining patterns as risk factors for intrahepatic vascular invasion (p:0.0400).

**Conclusion:**

Our results strongly suggest that overexpression of TXNIP has a pivotal role in HCC progression by inducing cell survival, invasion, and metastasis.

## INTRODUCTION

Hepatocellular carcinoma (HCC) is the second leading cause of cancer-related death worldwide [1]. Chronic infection with hepatitis B and/or hepatitis C viruses is the major cause of HCC. Other etiological factors include alcoholism, diabetes, and obesity. Aggressive nature of HCC result in a highly metastatic phenotype, poor prognosis in non-surgical patients and resistance against most traditional cancer therapies [2, 3].

Most cases of HCC develop in a background of fibrotic/cirrhotic liver. Independent from etiology, the common outcome of hepatocarcinogenesis is chronic inflammation associated with increased ‘‘reactive oxygen species (ROS)’’ levels [4, 5]. The elevated levels of ROS cause selective oxidative stress in tumor cells compared to normal cells. In addition, ROS promote carcinogenesis by inducing DNA damage and genetic instability [5]. Although many tumor cells can be eliminated by ROS, surviving tumor cells are forced to ‘‘cancer dormancy’’ with inhibited proliferation. During this stage, activation of redox-sensitive transcription factors in cancer cells can trigger a metabolic and phenotypic switch that results in increase of resistance to ROS [6–8]. This pleiotropic effect is regarded as an adaptive stress response to protect cells from increased ROS levels, leading to induction of epithelial-to-mesenchymal transition (EMT) and generation of more aggressive tumor foci via clonal expansion. In the meanwhile, cells can rearrange their cytoskeleton and repress adhesion molecules to promote motility [8, 9]. In the presence of high levels of ROS, the tumor microenvironment of HCC has been associated with increased recurrence rates and decreased survival [9]. However, little is known about how tumor cells regulate the levels of ROS and how changes in ROS levels influence cellular responses in HCC.

Thioredoxin-interacting protein (TXNIP), a redox-sensitive transcription factor, is characterized as a tumor suppressor gene and induced by cellular stress conditions. A large body of evidence suggests that elevated TXNIP expression elicits an increase in ROS levels, arrest in cell cycle, activation of apoptotic response and inhibition of glucose up-take in normal tissues [10–13]. In normal liver, spontaneous mutation of the TXNIP gene is associated with increased incidence of HCC [14].

Increased TXNIP expression levels are correlated with tumor growth inhibition in breast, thyroid and renal cancer models, suggestive of a tumor suppressor function in these tissues [15–17]. On the other hand, hyperglycemia-induced TXNIP overexpression is defined as a poor prognostic marker in pancreatic cancer [18]. In early HCC, increased TXNIP expression in well-differentiated, less motile and invasive HCC cell lines such as HuH-7, HepG2, and Hep3B is correlated with proliferation inhibition [19]. However, the role of TXNIP in tumor invasion and metastasis has not yet been addressed in HCC.

We and others have previously characterized various HCC cell lines as well- or poorly-differentiated. Poorly-differentiated HCC cell lines display mesenchymal phenotype with a highly motile and invasive character and deficient expression of hepatocyte lineage markers. However, well-differentiated cell lines share many features with hepatocytes and exhibit epithelial phenotype with limited motility and invasion capacity. Here, we show that TXNIP is expressed at very low levels in well-differentiated epithelial-like HCC cell lines. Surprisingly, poorly differentiated, highly motile and invasive HCC cell lines express significantly higher levels of TXNIP. Overexpression of TXNIP protects HCC cells from apoptosis, promotes EMT, migration, invasion and branching tubulogenesis while its silencing decreases cellular motility, invasion and ROS levels. Our data on larval zebrafish xenografts show that *TXNIP* overexpression promotes metastasis *in vivo*. Moreover, TXNIP expression in tumor samples from patients with primary HCC reveals that TXNIP levels are elevated in HCC tissues compared to normal liver tissues and adjacent non-tumor tissues. Finally, analyses of different datasets from the Oncomine database verify upregulation of the TXNIP in tumoral tissues as compared to their normal counterparts in HCC and other common cancers.

## RESULTS

### TXNIP is up-regulated in mesenchymal-like highly motile and invasive HCC cell lines and TXNIP expression positively correlates with ROS levels

**Figure 1:**
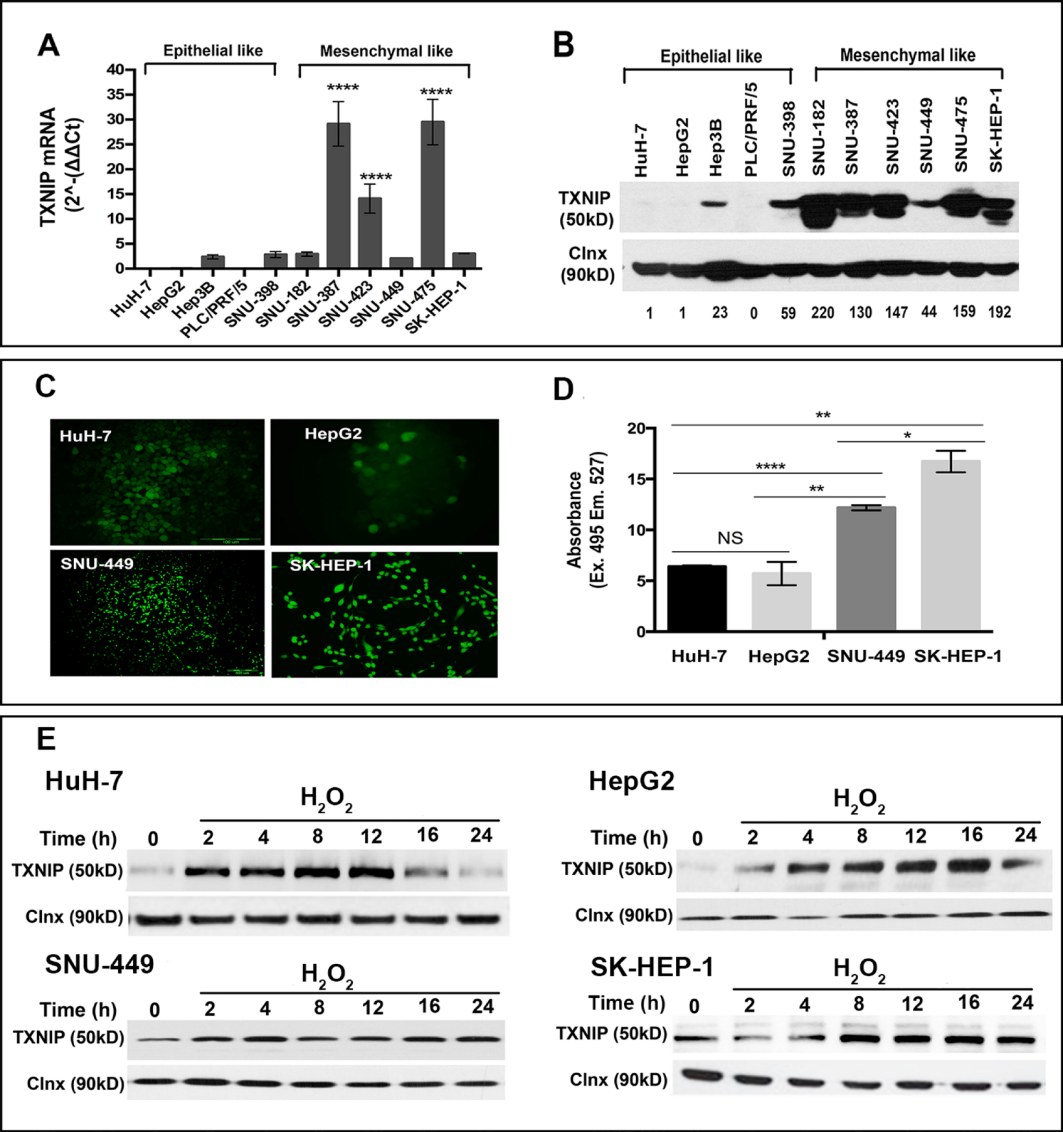
TXNIP expression levels in HCC cell lines (**A**, **B**) TXNIP expression in HCC cells was examined at transcriptional and protein levels by qPCR and Western Blotting (WB), respectively. Densitometric analysis of each band was done using ImageJ software. The minimum value was accepted as “1” and the others were rated based on it. Error bars ± SD (*n* = 3 experiments) ^****^p < 0.0001. (**C**, **D**) ROS levels of HuH-7, HepG2, SNU-449, and SK-HEP-1 were determined by DCFH-DA assay under basal conditions. Fluorescent emission was detected using fluorescence microscopy (left) and spectrofluorimetry (right). NS: not significant, ^*^p > 0.05, ^**^p < 0.01, ^***^p < 0.001, ^****^p < 0.0001. (**E**) TXNIP expression levels were determined under dose-dependent H_2_O_2_ treatment by WB in HuH-7, HepG2, SNU-449 and SK-HEP-1 cells.

Initially, we aimed to compare the levels of TXNIP expression in various epithelial- and mesenchymal-like liver cancer cell lines. TXNIP was absent or significantly reduced in less motile and invasive epithelial-like liver cancer cell lines HuH-7, HepG2 and PLC/PRF/5, while being up-regulated in highly motile and invasive lines SNU-182, -387, -423, -449, -475 and SK-HEP-1 at both mRNA (Figure 1A) and protein levels (Figure 1B). Next, we measured ROS levels of two HCC cell lines with no basal TXNIP expression (HuH-7, HepG2) and two liver cancer cell lines with high endogenous TXNIP expression (SNU-449 and SK-HEP-1) by fluorescent microscopy and spectrofluorometry. We observed that TXNIP expression positively correlated with ROS levels in all four cell lines (Figure 1C and 1D). Induction of intracellular ROS levels by H_2_O_2_ significantly increased TXNIP expression in a time-dependent manner (Figure 1E). Together, these data show that TXNIP expression positively correlates with the mesenchymal-like features and intracellular ROS levels of HCC cell lines.

### TXNIP overexpression causes a modest inhibition in cell cycle progression and protects HCC cells from apoptotic cell death

**Figure 2:**
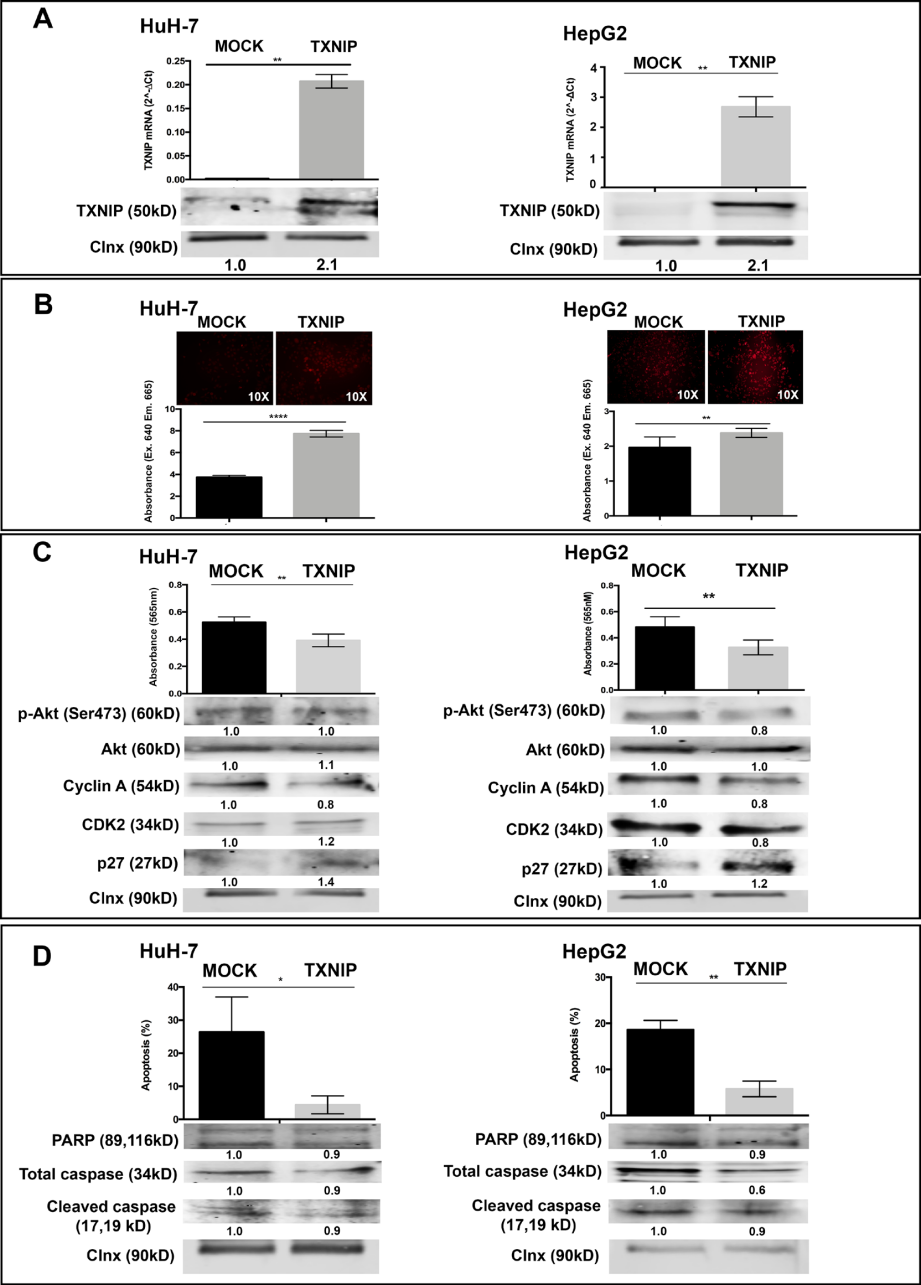
The effect of TXNIP overexpression on ROS levels, proliferation, apoptosis of HCC cells (**A**) TXNIP expression levels were determined by qPCR (top) and WB (bottom) in TXNIP overexpression vector and MOCK vector transfected HuH-7 and HepG2 cells. (**B**) The effect of TXNIP overexpression on ROS levels was analyzed by DCFH-DA assay using fluorescence microscopy (left) and spectrofluorimetry (right) read outs (**C**) Proliferation of MOCK and TXNIP transfected HuH-7 and HepG2 cells were detected by SRB assay (top). Absorbance levels were measured at 565 nm. Cell-cycle related molecules such as Cyclin A, CDK2 and p27 expression levels were detected by WB (bottom). (**D**) The effect of TXNIP overexpression on apoptosis was determined by Annexin V-FITC/PI double staining and quantified by flow cytometry (top). Apoptosis markers PARP, caspase and cleaved caspase expression levels were detected by WB (bottom). Calnexin was used as a loading control for WBs. Error bars ± SD (*n* = 3 experiments). ^*^p < 0.05, ^**^p < 0.01, ^***^p < 0.001, ^****^p < 0.0001.

To understand the functional role of TXNIP in HCC progression, we altered its expression in HCC cells by ectopic overexpression and small interfering RNA (siRNA) knockdown. Overexpression of TXNIP significantly increased ROS levels in both HuH-7 and HepG2 cells (Figure 2A, 2B). TXNIP overexpression weakly but significantly decreased proliferation of HuH-7 and HepG2 cells (Figure 2C, top panel) and likewise reduced activated Akt, Cyclin A and CDK2 levels (Figure 2C, bottom panel). Apoptosis was significantly decreased in TXNIP-overexpressing HuH-7 and HepG2 cells (Figure 2D, top panel). TXNIP overexpression caused a reduction in apoptosis-related molecules including PARP, caspase 3 and cleaved caspase 3, further supporting that TXNIP protects HCC cells from apoptosis (Figure 2D, bottom panel).

### TXNIP overexpression promotes EMT, migration, invasion and 3D branching tubulogenesis in HCC cells

**Figure 3:**
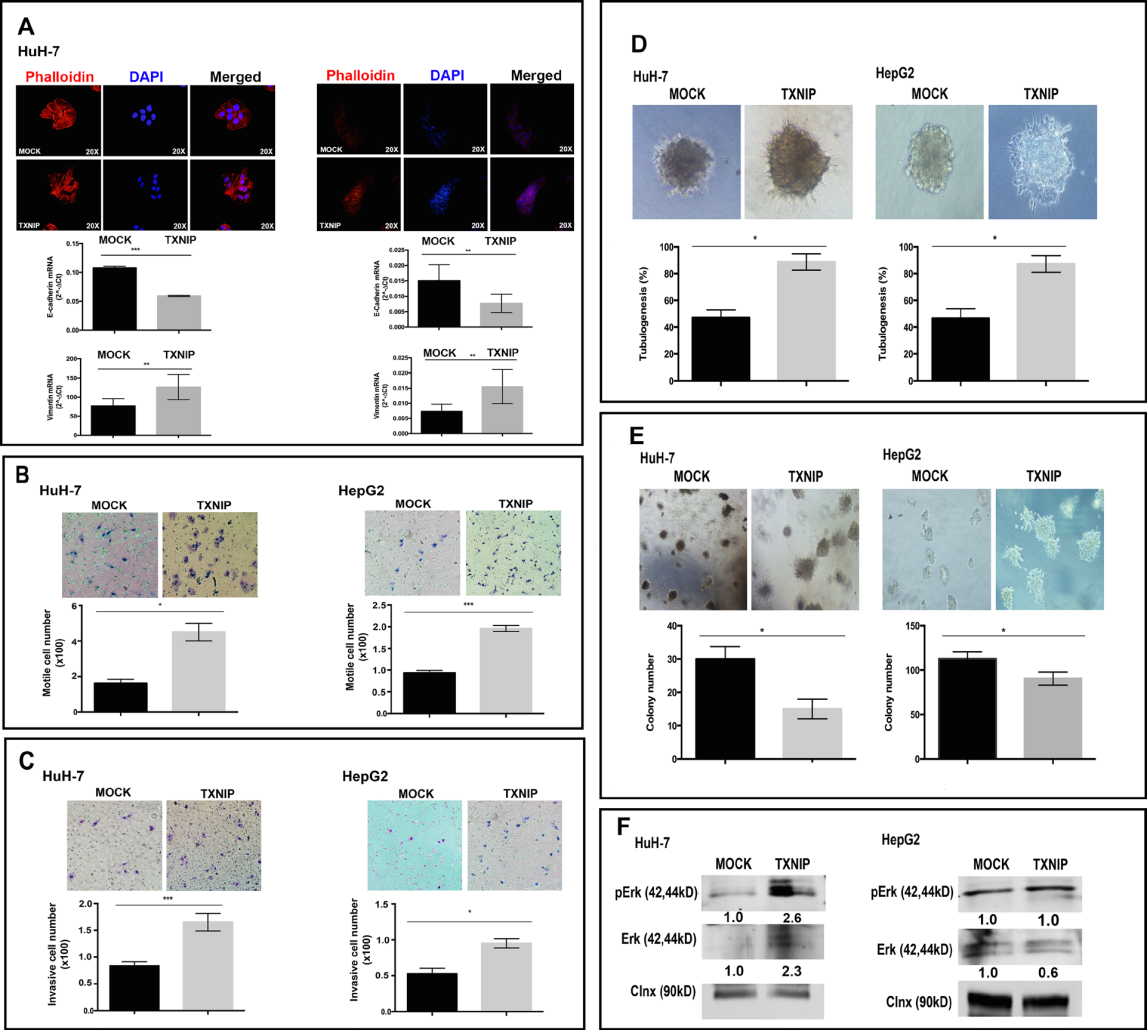
Regulatory roles of TXNIP overexpression on EMT, motility, invasion, branching tubulogenesis in HCC cells (**A**) Cytoskeletal changes associated with EMT were examined with Phalloidin staining in TXNIP and MOCK transfected HuH-7 and HepG2 cells. Cells were counterstained with DAPI. They were imaged by fluorescence microscopy (top). Expression levels of E-cadherin and Vimentin were determined by qPCR (bottom). (**B**, **C**) TXNIP and MOCK transfected HuH-7 and HepG2 cells were seeded into the upper chamber of Boyden chambers. The medium was added to the lower chamber. After 24 h incubation, the migrated cells were fixed, stained, counted and imaged by light microscope. For invasion assay, upper chamber was coated with Matrigel and invasive cell number was determined as described in the motility assay. Images show a representative experiment that had been performed in triplicate. Error bars ± SD (*n* = 3 experiments). ^*^p < 0.05, ^**^p < 0.01, ^***^p < 0.001. (**D**, **E**) TXNIP and MOCK transfected HuH-7 and HepG2 cells were seeded in 8-well chamber slide within collagen. After 14 days, colonies were counted under phase contrast microscopy and imaged. The results show the average number of colonies able to undergo branching morphogenesis and the average colony numbers formed per culture. Images show a representative experiment that had been performed in quadruplicate. (**F**) p-Erk1/2 and Erk1/2 expression were detected by WB in MOCK and TXNIP transfected HuH-7 and HepG2 cells. Densitometric analysis of each band was done using ImageJ software. The control conditions were accepted as “1” and the others were rated based on it. Error bars ± SD (*n* = 3 experiments) ^*^p < 0.05.

To determine the effects of TXNIP overexpression on F-actin fiber formation, TXNIP and MOCK transfected HuH-7 and HepG2 cells were stained with Phalloidin. TXNIP-overexpressing cells showed increased actin stress fibers (Figure 3A, top panel). Next, we found that expression of the prototypical epithelial cell marker E-cadherin was decreased whereas that of the mesenchymal marker Vimentin was increased at the transcriptional level in TXNIP overexpressing-cells (Figure 3A, bottom panel). Both qualitative and quantitative data showed that TXNIP overexpression significantly increased cellular motility (Figure 3B) and invasion (Figure 3C). Taken together, our results showed that overexpression of TXNIP promotes EMT, migration, and invasion of HCC cells. To understand the role of TXNIP on anchorage-dependent growth, differentiation, and morphogenesis of tumor cells, we performed branching morphogenesis assays. TXNIP overexpression induced sprouting and tubule formation in HCC cells whereas MOCK transfected cells mainly formed cysts (Figure 3D, top panel) and significantly promoted branching tubulogenesis in HuH-7 and HepG2 cells (Figure 3D, bottom panel). TXNIP-overexpressing cells formed fewer but larger colonies (Figure 3E). To test whether TXNIP can modify MAPK signaling pathway, which has critical roles in cell migration, invasion, and branching tubulogenesis, we examined the expression of p-Erk1/2 and Erk1/2. Erk1/2 expression and activation were indeed induced by TXNIP in HuH-7 cells (Figure 3F). These results indicate that TXNIP can enhance EMT, migration, invasion and 3D branching tubulogenesis in HCC cells.

### TXNIP silencing decreases ROS levels, cellular motility, and invasion of HCC cell lines

**Figure 4:**
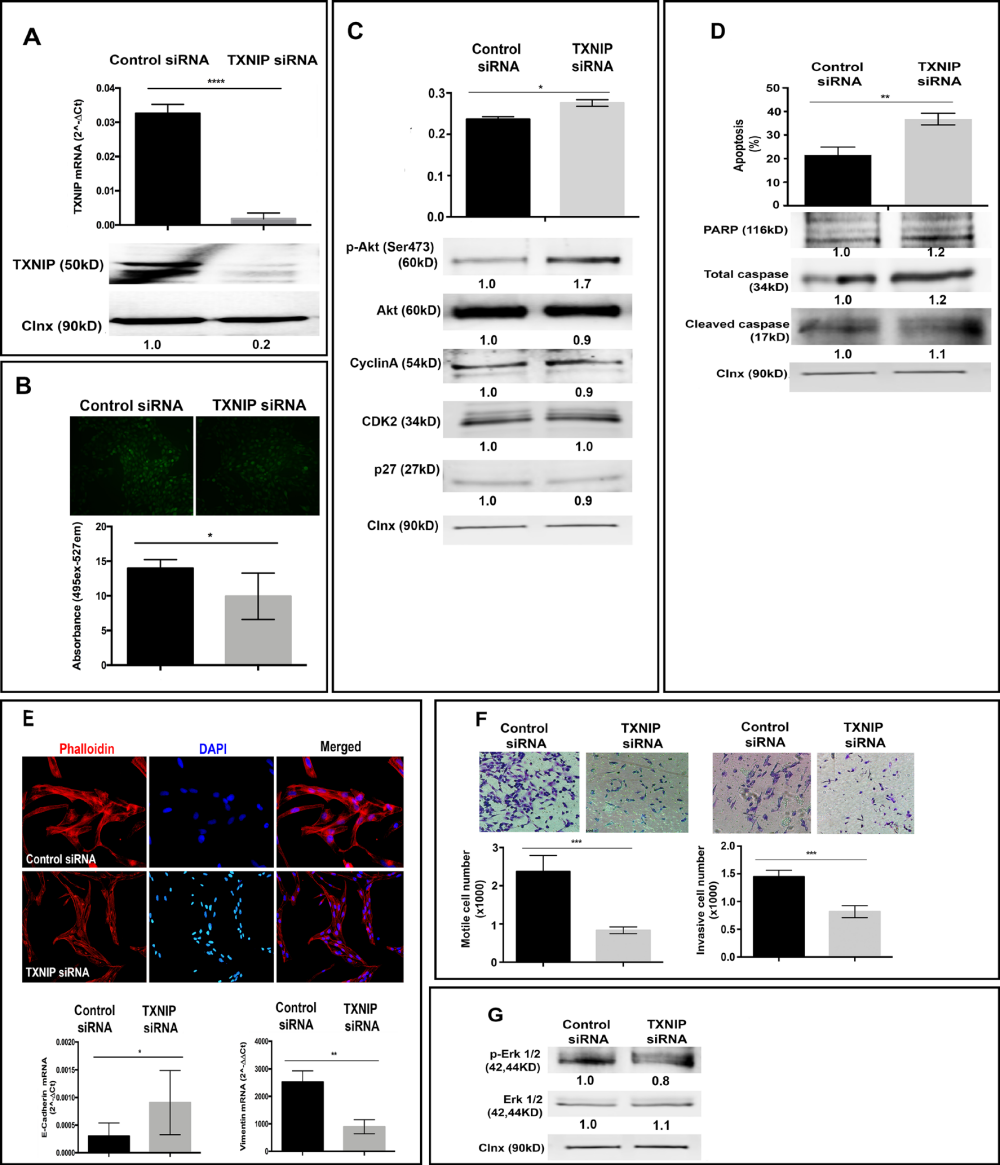
Effects of TXNIP silencing on cellular ROS levels, proliferation, apoptosis, EMT and invasion (**A**) TXNIP mRNA (top) and protein (bottom) expression levels were examined by qPCR and WB in TXNIP and control siRNA treated SK-HEP1 cells. (**B**) The effect of TXNIP silencing on ROS levels was detected as described above. (**C**) Proliferation levels of TXNIP and control siRNA treated SK-HEP1 cells were detected by SRB assay (top). Cyclin A, CDK2 and p27 expression levels were detected by WB (bottom). (**D**) The effect of TXNIP silencing on apoptosis was determined by Annexin V-FITC/PI double staining and quantified by flow cytometry (top). In addition, apoptosis markers PARP, caspase and cleaved caspase expression levels were determined by western blotting (bottom). (**E**) Cytoskeletal changes associated with EMT were determined with Phalloidin staining in TXNIP siRNA and control siRNA treated SK-HEP1 cells. Cells were counterstained with DAPI and, imaged by fluorescence microscopy (top). Expressions of E-cadherin and Vimentin were analyzed by qPCR (bottom). (**F**) To determine the effects of TXNIP silencing on motility (left) and invasion (right) Boyden chamber assay was used. The cells that migrated and invaded through the membrane were stained, imaged and counted under a light microscope. The results are representative of three independent experiments, done in quadruplicate. The results represent the mean number of migrated and invaded cells per well. (**G**) p-Erk 1/2 and Erk 1/2 expressions were determined by WB. Calnexin was used as a loading control for WBs. Densitometric analysis of each band was done by ImageJ software. The minimum value was accepted as “1” and the others were rated upon it. Error bars ± SD (*n* = 3 experiments). ^*^p < 0.05, ^**^p < 0.01, ^***^p < 0.001, ^****^p < 0.0001.

Next, to test whether TXNIP inhibition could reverse the phenotypes observed in TXNIP overexpression, we exploited siRNA-mediated knockdown of gene expression. Transfection of SK-HEP-1 cell line with TXNIP siRNA resulted in dramatic reduction of TXNIP expression compared to control siRNA both at mRNA (Figure 4A, top panel) and protein levels (Figure 4A, bottom panel). TXNIP silencing significantly decreased intracellular ROS levels (Figure 4B) and p27 expression (Figure 4C, bottom panel), while increasing cell proliferation (Figure 4C, top panel), Akt activation and expression (Figure 4C, bottom panel). Likewise, SK-HEP-1 cells silenced for TXNIP expression exhibited significantly increased apoptotic cell death (Figure 4D, top panel), also marked by PARP, caspase 3 and cleaved caspase 3 (Figure 4D, bottom panel). TXNIP-silenced cells displayed mesenchymal-to-epithelial transition (MET), allowing cells to reverse EMT (Figure 4E, top panel) and elevated levels of the epithelial cell marker E-cadherin (Figure 4E, bottom panel). TXNIP silencing significantly decreased cellular motility (Figure 4F, left panel) and invasion (Figure 4F, right panel). In addition, p-Erk1/2 level was decreased in cells treated with TXNIP siRNA (Figure 4G). Of note, we obtained similar results from the experiments performed in SNU-449 cell line (Supplementary Figure 1). These phenotypes caused by TXNIP siRNA were efficiently rescued by TXNIP overexpression (Supplementary Figure 2). Thus, we conclude that inhibition of TXNIP expression is sufficient to reduce intracellular ROS levels, cellular motility, and invasion while inducing apoptotic cell death in HCC cell lines.

### TXNIP overexpression induces metastasis in zebrafish embryos

**Figure 5:**
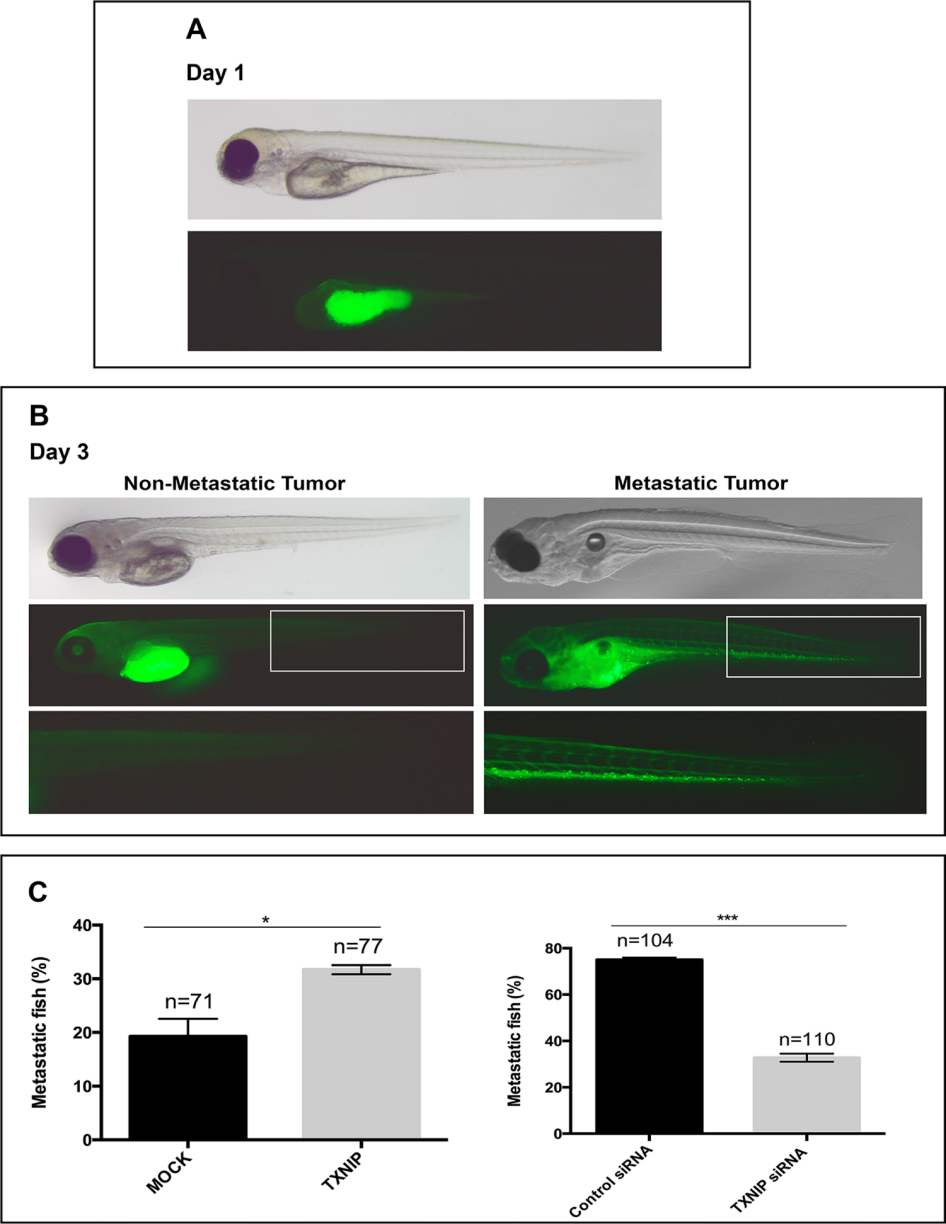
Regulatory effects of TXNIP on metastatic ability of HCC cells in zebrafish embryos MOCK or TXNIP transfected HepG2 cells and control or TXNIP siRNA treated SNU-449 cells were used. These cells were microinjected in the yolk sac of 2dfp (days post fertilization) zebrafish embryos (Day 0). (**A**) At 20–24 hpi (hours post injection), the embryos that harbored an HCC cell mass in the yolk were selected (Day 1). For quantification of metastasis, fluorescent (bottom) and bright field (top) images were captured (**B**). The number of embryos with metastasis was counted by fluorescent microscopy at day 3 dpi (days post injection) (**C**). Error bars, ± SD (*n* = 2 experiments) ^*^p < 0.05, ^***^p < 0.001.

To test the effects of TXNIP on HCC cell metastasis *in vivo*, we performed xenograft experiments using zebrafish embryos as host [20, 21]. HepG2 cells were used to test the capacity of TXNIP overexpression in inducing metastatic behavior of HCC cells *in vivo*. We also used SNU-449 cells to test whether TXNIP suppression could repress metastasis. When 200–300 HCC cells were injected into the central part of the yolk sac of 48 hours-post-fertilization (hpf) zebrafish embryos, they formed a microscopically detectable mass within the yolk. Initially, by observing the xenografts at 4 hours-post-injection (hpi), we sorted out the embryos that did not receive any HCC cells, or exhibited cells dispersed within yolk and/or in circulatory system. At 20–24 hpi, we selected the embryos that harbored an HCC cell mass in the yolk, with no HCC cells in other parts of the organism, to ensure that the analysis was performed on local tumors of similar size (Figure 5A). At 3 days-post-injection (dpi), we classified the tumors based on metastasis: the xenografts with HCC cells in the trunk and/or head of the embryos were considered as metastatic, whereas those with a single mass of HCC cells in yolk as non-metastatic (Figure 5B). We found that MOCK transfected HepG2 cells metastasized to tail/head of the embryo in 18% of the xenografts, while TXNIP transfected HepG2 cells metastasized in 30% of the xenografts (Figure 5C, left panel). On the other hand, when control SNU-449 cells were injected into yolk sac, 75% of the xenografts displayed metastatic behavior (Figure 5C, right panel) and this was significantly reduced to 32% when TXNIP was silenced with siRNA in SNU-449 cells (Figure 5C, right) Collectively, our data suggest that TXNIP is a potent inducer of metastasis in HCC cells *in vivo*.

### TXNIP expression is higher in HCC tumoral tissues than in adjacent non-tumoral, normal and cirrhotic liver tissues

**Figure 6:**
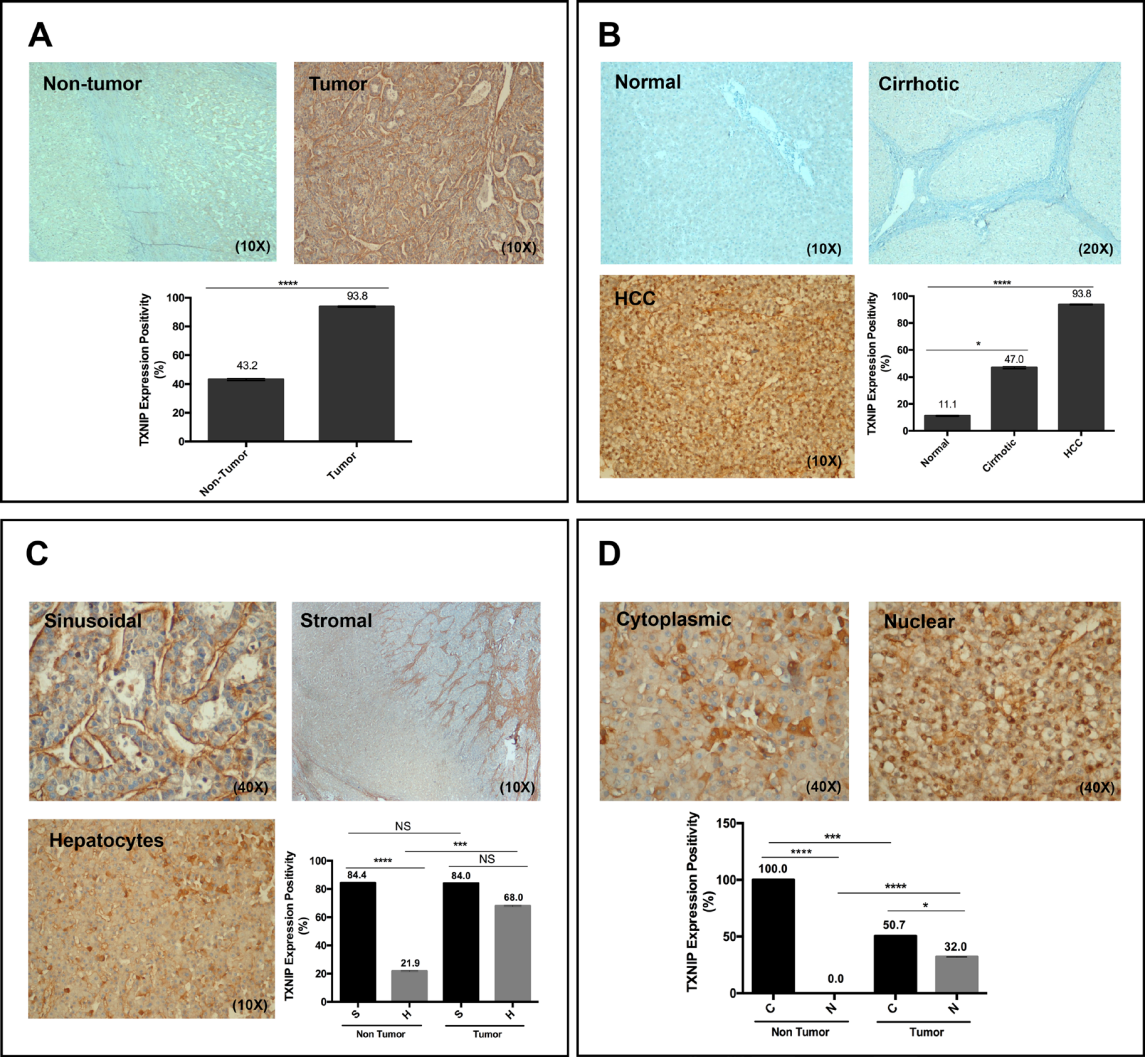
TXNIP levels in normal, cirrhotic and HCC tumor samples (**A**, **B**) TXNIP expression levels were determined in both primary HCC tumor samples and their adjacent non-tumor tissue as well as in normal and cirrhotic liver tissues by IHC analysis. (**C**, **D**) TXNIP staining patterns were analyzed in primary HCC tumor tissues and surrounding non-tumoral tissues. NS: not significant, ^*^p > 0.05, ^***^p < 0.001, ^****^p < 0.0001.

To compare the levels of TXNIP expression in HCC tumoral and non-tumoral tissues, we examined TXNIP expression in 80 HCC tumoral and adjacent non-tumoral tissues of 73 cases (66 cirrhotic, 7 non-cirrhotic cases) as well as in normal liver samples (*n* = 11). TXNIP expression was significantly higher in HCC tumoral areas (93.8%) than in their non-tumoral counterparts (43.2%) (Figure 6A). We detected TXNIP expression only in 1 out of 11 (11.1%) normal liver tissues (Figure 6B). In addition, among the adjacent non-tumor liver tissues tested, 31 out of 66 (47.0%) adjacent cirrhotic liver tissues and 1 out of 7 (14.3%) non-cirrhotic liver tissues expressed TXNIP. TXNIP expression was observed in the cytoplasm or nuclei of the hepatocytes as well as in the sinusoids (sinusoidal pattern) and/or stroma (stromal pattern) adjacent to the hepatocytes. On the other hand, hepatic sinusoids of the normal liver samples were negative for TXNIP expression. We observed a sinusoidal/stromal staining pattern in 63 out of 75 (84.0%) TXNIP positive HCC tumoral tissues and in 27 out of 32 (84.4%) adjacent non-tumoral tissues (Figure 6C). TXNIP expression in hepatocytes, however, was significantly higher in TXNIP positive HCC tumoral tissues (68%) than in non-tumoral counterparts (21.9%) (Figure 6C). By comparing TXNIP expression pattern between adjacent tumoral and non-tumoral tissues, we detected nuclear TXNIP staining only in tumoral tissues and cytoplasmic TXNIP only in non-tumoral ones (Figure 6D). These findings indicate an elevation of TXNIP expression levels in HCC tumoral tissues as compared to their non-tumoral counterparts.

### TXNIP overexpression is positively correlated with viral infection and invasion in HCC patients

Etiological stratification revealed that overall TXNIP expression was higher in viral infection- (both HCV and HBV) related HCCs than non-viral cases (p:0.0001). Sinusoidal/stromal TXNIP expression in HCC cases positively correlated with intrahepatic vascular invasion away from the tumor (p:0.0400). Cytoplasmic TXNIP expression was higher in cases with viral etiology (p:0.0040) and positively correlated with portal vein invasion (p:0.0480). Interestingly, nuclear TXNIP expression was significantly higher in male HCC patients (p:0.0400) and in HCC patients with viral etiology (p:0.0001). Clinicopathological features of these patients and their levels of TXNIP expression in liver tissues are documented in Table 1.

**Table 1:** The clinicopathological features of HCC patients and TXNIP expression in liver samples

Parameters	Variable	No of patients (*n* = 80)	
		*n*	%
**Gender**	Male	67	83.8
	Female	13	16.2
**Alcohol intake**	Present	13	16.2
**Viral infection**	Present	72	90.0
**Portal vein invasion**	Present	12	15.0
**Out of tumor invasion**	Present	15	18.8
**Tumoral invasion**	Present	16	20.0
**Reccurrence**	Present	12	18.2
**Nodule number**	>3	27	33.8
**Tumor diameter**	<2 cm	66	82.5
**Differentiation**	Well	16	20.0
	Moderate	43	53.8
	Poor	13	16.2
	ND	8	10.0
**Adjacent tissue**	Cirrhotic	66	88.5
**Nuclear degree**	1	7	8.9
	2	21	26.6
	3	43	54.4
	4	8	10.1
**TXNIP Positivity**	Normal liver (*n* = 11)	1	11.1
	Non-tumor cirrhotic liver (*n* = 66)	31	47.0
	Non-tumor non-cirrhotic (*n* = 7)	1	14.3
	Tumor (*n* = 80)	75	93.8

**Figure 7:**
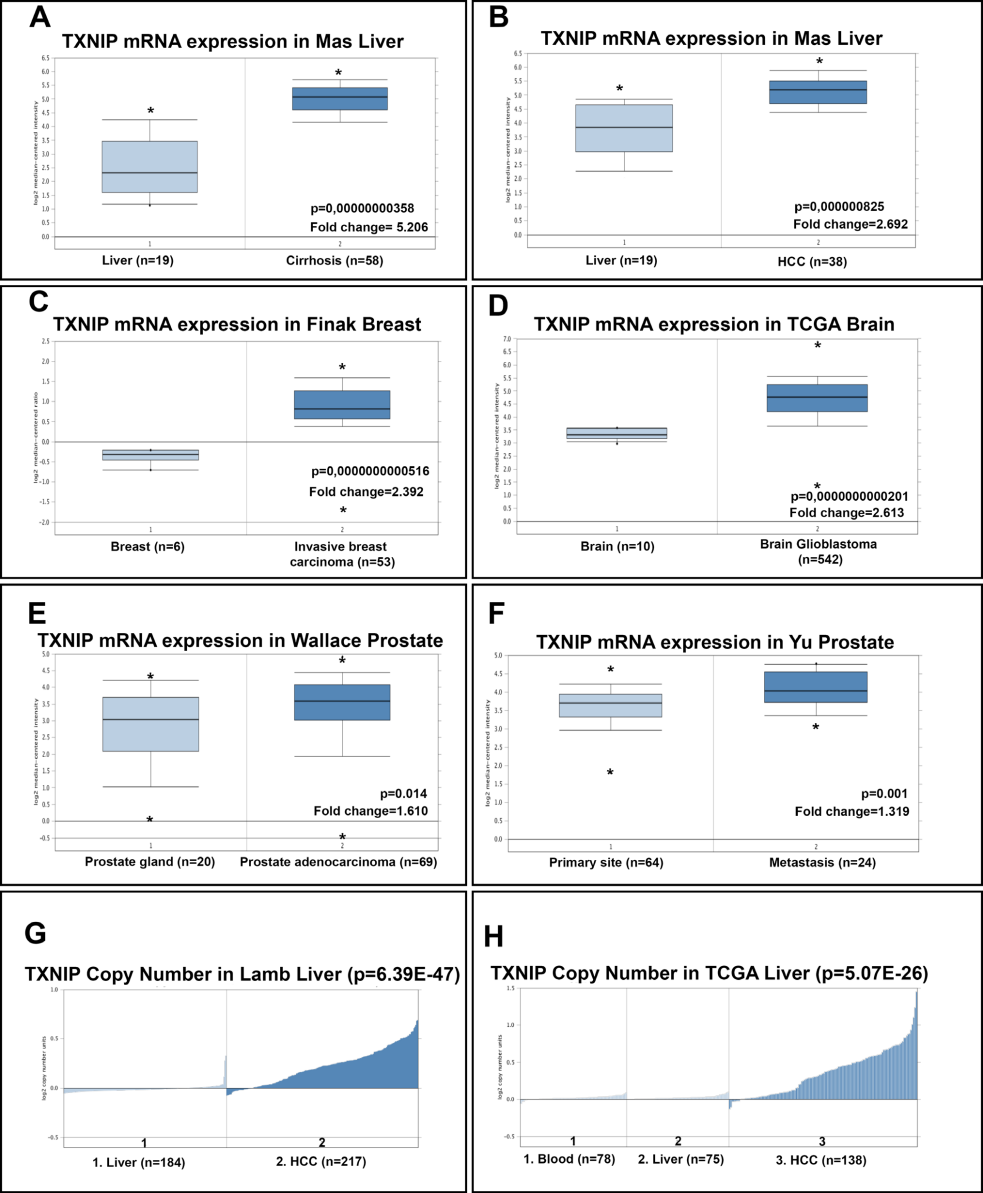
Oncomine analysis of TXNIP mRNA levels and copy number of TXNIP gene in cancer versus normal tissues Oncomine data mining analysis of TXNIP mRNA levels in (**A**, **B**) Mas Liver: normal liver vs cirrhotic liver and normal liver vs. HCC, (**C**) Finak Breast: invasive breast carcinoma vs normal breast samples, (**D**) TCGA Brain: brain glioblastoma vs. normal brain samples, (**E**) Wallace Prostate: prostate adenocarcinoma vs. prostate gland, (**F**) Yu Prostate: metastatic cancer vs. primary tumor. TXNIP gene copy number in (**G**) Lamb Liver: normal liver vs HCC, (**H**) TCGA liver: Blood vs liver vs HCC.

Next, we analyzed seven independent microarray datasets and The Cancer Genome Atlas (TCGA) datasets using Oncomine Platform (Figure 7A–7H) [22]. We found that mRNA levels of TXNIP in Mas Liver [23] are consistent with our data (Figure 7A and 7B). Our analysis of copy number alteration data from Lamb [24] and TCGA liver data sets in Oncomine (https://www.oncomine.org) revealed that TXNIP copy number was significantly higher in HCC tumoral tissues than in normal liver (Figure 7G and 7H). Finally, TXNIP expression in tumoral tissues of especially invasive and metastatic cancers such as breast [25], brain (TCGA-Glioblastoma Multiforme (GBM) Gene Expression Data, in Oncomine (https://www.oncomine.org)) and prostate [26, 27] cancers were increased as compared to their non-tumoral counterparts. Thus, we conclude that high levels of TXNIP in tumoral tissues might be common among invasive and metastatic cancers.

## DISCUSSION

Tumor metastasis is responsible for the majority of cancer deaths, and EMT plays crucial roles in metastasis. Many examples support the role of increased ROS levels in the activation of EMT and the metastatic phenotype [5–7]. A common feature of hepatocarcinogenesis is that chronic hepatic inflammation, regardless of etiology, results in elevation of intracellular ROS [4]. Several studies have revealed the importance of TXNIP in regulation of intracellular ROS levels and that TXNIP-mediated increase in ROS results in proliferation inhibition and further ROS elevation [28, 29]. In normal liver, TXNIP deficiency was found to be sufficient for development of hepatic tumors in relevant mouse models [18, 19]. TXNIP expression is low or absent in well-differentiated HCC cell lines characterized by low motility and invasiveness [30–34]. Moreover, a large body of evidence supports the role of TXNIP as a tumor suppressor in several cancer types [14–16, 35, 36]. Conversely, some studies showed that increase in TXNIP expression is an important switch in response to stress conditions, such as oxidative stress, hypoxia and lactic acidosis, to induce trans-epithelial migration and metastasis of tumor cells [9, 37–40]. Nevertheless, little is known about the mechanisms by which TXNIP regulate cell invasion and metastasis in HCC. This study uncovers the role of TXNIP in progression of HCC by using primary liver tissues, zebrafish xenografts and HCC cell lines. Our results suggest that TXNIP expression is low in well-differentiated HCC cell lines, whereas it is selectively high in poorly differentiated, highly motile and invasive HCC cell lines. [31, 34, 39, 40, 41].

**Figure 8:**
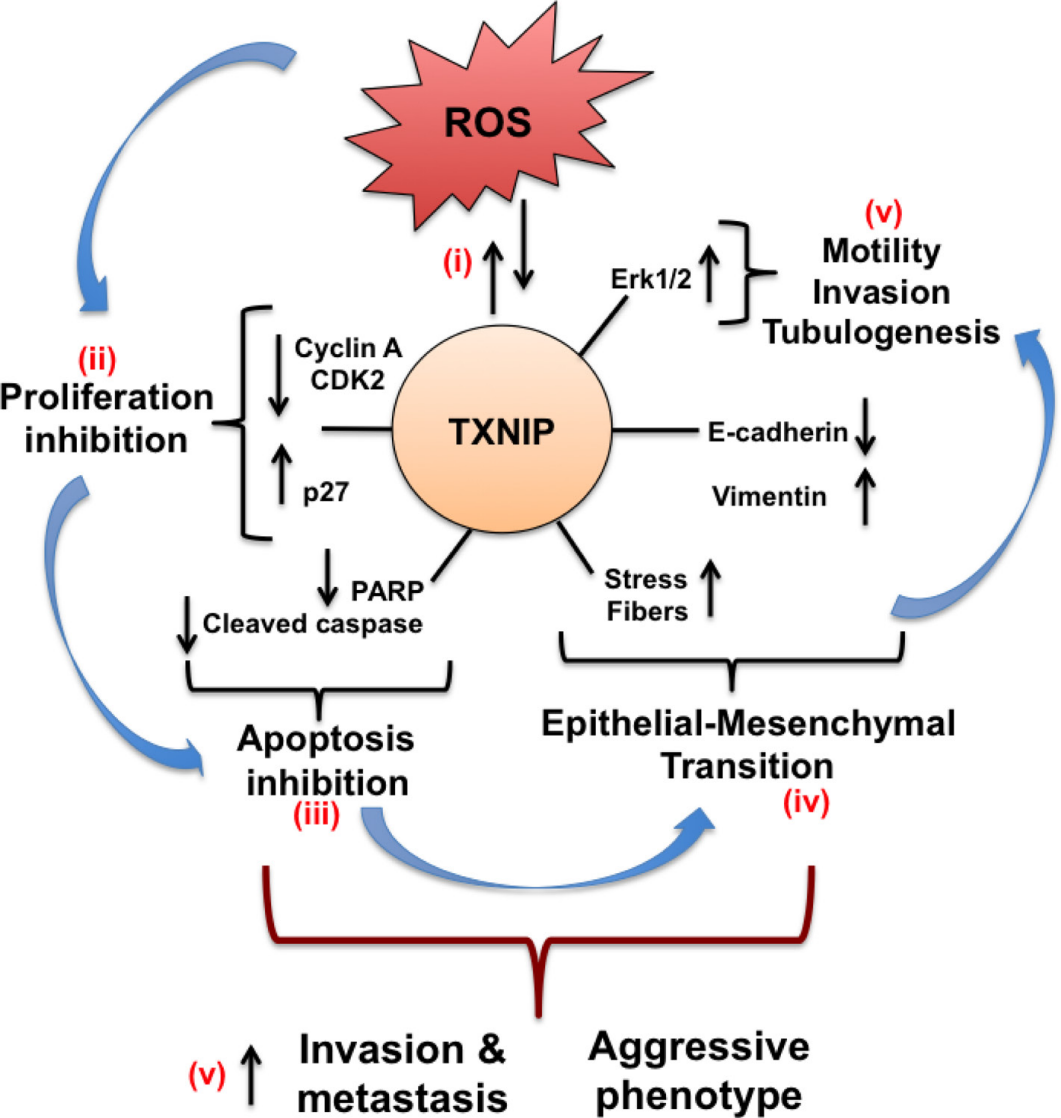
Schematic representation of the effects of TXNIP overexpression in HCC cells (i) Cellular ROS increment strongly induces TXNIP expression. Through a self-regulating mechanism, TXNIP promotes its own expression via upregulating cellular ROS levels. (ii) TXNIP overexpression, in turn, inhibits cell growth and proliferation by inducing CDK2, CyclinA repression, and p27 up-regulation. (iii) Moreover, TXNIP modulates apoptosis by regulating PARP and cleaved caspase levels and protects HCC cells from apoptotic cell death. (iv) TXNIP overexpression further alters E-cadherin and Vimentin transcription, induces stress fiber formation and promotes EMT. (v) Eventually, TXNIP triggers aggressive phenotype by increasing cell motility, invasion and branching tubulogenesis.

Our data support the following mechanism for TXNIP function in HCC progression (Figure 8): (i) Elevation of intracellular ROS strongly induces TXNIP, which in turn promotes its own expression via upregulating ROS levels; (ii) TXNIP inhibits cell growth and proliferation by inducing p-Akt, CDK2, p27 and repressing Cyclin A; (iii) TXNIP promotes cell cycle arrest in HCC cells and protects them from apoptotic cell death by decreasing PARP and cleaved caspase levels; (iv) TXNIP further alters E-cadherin and Vimentin expression, induces stress fiber formation and promotes EMT; and finally (v) TXNIP triggers aggressive HCC phenotype via increasing cell motility, invasion, branching tubulogenesis and Erk1/2 activation.

Ectopic overexpression of TXNIP, similar to H_2_O_2_ treatment, causes a significant increase in ROS levels and induces motility, invasion and anchorage-dependent growth in HCC cells associated with increasing Erk1/2 activation. Moreover, we observe a positive correlation between ROS levels and TXNIP expression in all HCC cell lines we tested. ROS levels are high in poorly differentiated, highly invasive and metastatic, mesenchymal-like HCC cell lines that have high TXNIP expression. Besides, high TXNIP levels result in EMT phenotype with decreased epithelial markers and increased mesenchymal markers. TXNIP-overexpressing cells change their cuboid, epithelial-like shape to a spindle, fibroblastic-like appearance.

TXNIP overexpression moderately inhibits proliferation in all tested HCC cell lines, supporting the previous reports. However, here we show that high levels of TXNIP does not promote apoptotic cell death, evidenced by decreased annexin-V, cleaved caspase, and PARP levels. Rather, cell cycle arrest induced by TXNIP appears to protect HCC cells from apoptotic cell death through inhibition of CDK2, Cyclin A and Akt activation. Since inhibition of proliferation by Akt signaling induces “cancer dormancy” and protects cancer cells from ROS induced apoptosis [41, 42], TXNIP likely plays a role in selection of resistant clones.

Our results support a promoting role for TXNIP in Erk1/2 activation in HCC cells. Similarly, hyperglycemia-induced TXNIP can activate p38 and Erk1/2 MAPK pathways in pancreatic cancer model and TXNIP expression is positively correlated with poor prognosis in pancreatic cancer patients [17]. These findings together suggest that elevated levels of TXNIP and Erk1/2 phosphorylation and decreased Akt activation collectively cause a resistance to proliferation inhibition and selection of resistant clones in HCC.

We validate the importance of TXNIP overexpression in HCC progression with clinical samples, zebrafish xenograft metastasis model and Oncomine dataset analysis. TXNIP expression is significantly higher in primary HCC tumoral tissues than in normal or cirrhotic liver as well as in non-tumoral adjacent tissues. Similarly, mRNA and protein expression levels of TXNIP have been found to be higher in the tumoral tissues of pancreatic cancer patients with diabetes [18]. A recent work has reported that TXNIP expression is elevated in HBV associated-HCC tissues, suggesting that TXNIP overexpression is an independent risk factor for metastasis in HCC [43]. Etiological stratification of our HCC patients displays a similar pattern where overall TXNIP expression is significantly higher in viral infection- (both HCV or HBV) related HCCs than in non-viral cases. Our analysis of intracellular distribution of TXNIP in primary liver tissues has been a pioneering study, revealing that TXNIP expression is higher in the nuclei and lower in the cytoplasm of HCC tumoral tissues than in adjacent non-tumoral tissues. Since TXNIP expression has mostly been determined quantitatively by qPCR or Western blotting in the previous reports, we believe that qualitative analysis of subcellular distribution of TXNIP in normal, cirrhotic and HCC tissues will provide mechanistic insight into its functional role. Future studies will clarify the subcellular localization-dependent effects of TXNIP in HCC progression.

## MATERIALS AND METHODS

### Clinical samples, tissue processing, and ethical considerations

A total of 80 HCC patients who underwent liver transplantation or resection in Ege University, Izmir, Turkey were included in this study. Before surgery, none of the patients received any treatment. Demographic, laboratory and clinical data were obtained from patient files and their relationship with histopathological data was evaluated. Normal liver tissues (*n* = 11) were obtained from living donors or from patients who underwent resection due to trauma. The study was approved by the Ethics Committee of Dokuz Eylul University Medical School (Number: 2016/22–32). Written informed consents were obtained from patients before the operations or liver biopsy procedures.

### Histopathological examination

All tissue samples were fixed in formalin, processed with conventional methods and embedded in paraffin [44]. Tissue sections were reevaluated by a certified pathologist (FY) for the confirmation of the diagnosis and the selection of the most appropriate tissue block for immunohistochemistry. Determination of the differentiation status of the tumors was made according to the 2010 classification of World Health Organisation [45] and the grading of the tumors was based on Edmondson-Steiner grading system [46]. For each case, the number of HCC nodules, total tumor diameter, largest tumor diameter, average tumor diameter, tumor differentiation (well, moderate, poor), vascular invasion (inside/adjacent/distant to the tumor), portal vein invasion, nuclear grade (Edmondson Grades 1, 2, 3, 4) were determined for further analysis.

### Immunohistochemical procedure

Immunohistochemical analyses were performed on 4-μm-thick sections taken on lysine-coated slides. Sections were deparaffinized and then rehydrated. Immunohistochemical staining for Anti-TXNIP antibody (Sigma-Aldrich, HPA031085) was performed at 1:100 dilution using an automated immunohistochemical stainer according to the manufacturer's guidelines (streptavidin-peroxidase protocol; BenchMark, Ventana, USA). The sections were then stained with 3, 3-diaminobenzidine tetrahydrochloride (DAB), a chromogen stain (brown in color), and counterstained with hematoxylin.

### Evaluation of staining

All staining was semi-quantitatively evaluated by a certified pathologist (FY). The blinded evaluation was performed for histopathological analysis. Staining with TXNIP antibody was defined as cytoplasmic and/or nuclear staining in hepatocytes and sinusoidal/stromal staining within the tumor. Cytoplasmic staining was limited to the cytoplasm of the cells, whereas nuclear staining was only in the nuclei. Sinusoidal/stromal staining was either a linear staining along the sinusoids or, when present, was together with a more diffuse staining in the stroma of the tumor. The extent of the staining pattern was again scored semi-quantitatively.

### Animal studies

Zebrafish were reared under standard conditions in a Zebtech multilinking system. Animals received humane care, the “Guide for the Care and Use of Laboratory Animals” prepared by the National Academy of Sciences and published by the National Institutes of Health was followed. 2-dpf-5dpf zebrafish embryos of Golden strain were used. Gender of the species is unknown at this stage, and animals do not eat food. Adult zebrafish were fed with the standard diet composed of live artemia and flakes. Xenograft injections were done during the light cycle.

Zebrafish was used as a host for xenograft studies as described before [20, 21]. Briefly, HCC cells were labeled with 2 mg/ml DiO (Molecular Probes, V22886). Labeled cells were re-suspended to a final density of 30,000 cells/μl. Phenol red (Sigma, P0290) was used for visibility of cell suspension during injection. Zebrafish were reared under standard conditions in a ZebTec multilinking system. Embryos of wild-type Golden strain were dechorionated at 48 hours post fertilization (hpf) with 1 mg/ml pronase (Sigma, P5147), rinsed with embryo medium (E3 medium) and anesthetized with Tricaine. Embryos were placed laterally on 1% agarose prepared with an injection mold. 10 μl of cell suspension was transferred into the center of the yolk sac, using a capillary needle attached to a micromanipulator (Narishige MN-151) and an injector (PV820 pneumatic picopump, World Precision Instruments). Embryos were incubated at 35° C. Xenografts were examined at 4 hpi and the embryos that did not receive any HCC cells or had cells dispersed within yolk or in the embryo circulatory system were separated and excluded from the study. At 20–24 hpi, the embryos that harbored an HCC cell mass in the yolk, and did not have HCC cells in other parts of the organism, were included to the study. Xenografts were examined in a blinded manner under an Olympus SZX16 Fluorescent Stereomicroscope equipped with XC50 camera.

### Cell culture

Human liver cancer cell lines: HuH-7, HepG2, Hep3B, PLC/PRF/5, SNU-398, SNU- 182, SNU-387, SNU-423, SNU-449, SNU-475 and SK-HEP-1, were cultivated as described previously [47]. To exclude mycoplasma contamination, all cell lines were regularly tested with PCR based mycoplasma detection kit (EZ-PCR Mycoplasma Test Kit, Biological Industries, 20-700-10). Authentication of cells was done by DNA profiling at the University of Colorado Cancer Center (UCCC) (UCCC) DNA Sequencing & Analysis Shared Resource (CO, USA) using Applied Biosystem's Identifiler kit (PN 4322288).

### Generation of stable cell lines

A retroviral vector TTI-GFP-TXNIP expressing TXNIP was constructed by sub-cloning cDNA of TXNIP from the p-CMV-6AC-TXNIP vector, which was purchased from Origene (RG210804). Empty TTI-GFP vector was used as a control. rtTA3-hygro retroviral vector was used to generate inducible Tet-On system. After cloning studies, target HuH-7 and HepG2 cells were stably transduced as reported previously [48, 49].

### Silencing and rescue of TXNIP expression by small-interfering RNA transfection

SNU-449 and SK-HEP-1 cells were transfected with 1 μM TXNIP or non-targeting siRNA (Accell Smart pool, E-010814-00, D-001910-01, respectively Dharmacon, UK) according to the manufacturer's instructions. SK-HEP-1 and SNU-449 cell lines were used for rescue experiments. Briefly, cells were initially treated with TXNIP siRNA for 24 hours, then siRNA delivery media was removed and cells were transfected with the TXNIP over-expression and MOCK vectors, TTI-GFP-TXNIP and TTI-GFP-MOCK, respectively. Efficiency of endogenous TXNIP silencing and exogenous TXNIP over-expression was analyzed by qPCR and Western blotting.

### Real-Time RT-PCR

The total RNA was extracted using a TRIZOL reagent (Sigma, UK). Total RNA was reverse-transcribed into cDNA with synthesis kit (Thermo, K1671, US) according to manufacturer's instructions. Power SYBR Green PCR Master Mix (Life Technologies, 4368706, USA) in StepOnePlus Real-Time PCR System (Applied Biosystems, USA) was used for qPCR analysis. RPL41 expression was used as internal control. TXNIP, E-cadherin, and Vimentin expressions were normalized to RPL41 using the 2−ΔCt method. Primers used in qPCR reactions are presented in Supplementary Table 1.

### Western blotting

Total protein was prepared as described previously [50]. Antibodies against TXNIP (K-0203-5, MBL, Japan), Cyclin A, CDK2, p27, Erk 1/2, Calnexin (Santa Cruz, 239, 6248, 1641, 93, 11397, US, respectively), phosho-42/44 MAPK (Thr202/Tyr204), PARP, Caspase-3 (Cell signaling 19G2, 7150, 9662, respectively) were used for western blotting experiments as described previously [35, 40, 49]. Equal loading and transfer were confirmed by repeat probing for calnexin. Band intensities were quantified as pixels by using ImageJ software (NIH).

### Sulphorhodamine B assay

Cell proliferation was examined by Sulphorhodamine B (SRB) assay and xCelligence real-time cell analysis system as described previously [49, 50].

### Annexin-V apoptosis assay

The Annexin V-PE Apoptosis Detection kit (Biovision, K128, US) was used to analyze apoptosis, according to the manufacturer's instructions. Cisplatin (10 μM) was used as a positive control. Cells were analyzed by flow cytometry (BD, FacsCanto II) and results were analyzed with BD CellQuestPro software.

### Phalloidin staining

For staining of the actin filaments, tetramethylrhodamine–phalloidin (Sigma P1951, UK) was used according to the manufacturer's instructions. Cells were visualized using an Olympus BX50 fluorescence microscope (Olympus, Tokyo, Japan).

### Motility and invasion assay

*In vitro* motility and invasion assays were performed as described previously [47, 49]. Cells had traversed through the membrane were counted for each chamber using a bright-field inverted microscope. Measurements were performed for at least three technical and two biological replicates.

### Branching-morphogenesis assays

Cells were embedded in three-dimensional collagen I gels (BD 354236, US) as previously described [51]. For the quantitation of the morphogenic response, both size and number of colonies, as well as a number of branches per experimental condition were analyzed by using Olympus (US) CKX41 microscope. Data were expressed as the mean ± standard deviation (SD) for at least four independent experiments.

### Oncomine and the cancer genome atlas (TCGA) data analysis

A series of microarray datasets were retrieved from the Oncomine database (https://www.oncomine.org) to investigate TXNIP mRNA expression level in cancer [22]. The mRNA expression level and copy number variations (CNVs) of TXNIP gene from different cancer datasets were compared. Three HCC, one breast, one brain and two prostate cancer datasets including Mas *et al.* [23], Lamb *et al.* [24], TCGA liver and GBM datasets in Oncomine (https://www.oncomine.org), Finak *et al.* [25], Wallace *et al.* [26] and Yu *et al.* [27] were included in the study. The differential expression and CNVs for TXNIP gene between tumor and normal tissues were analyzed and their fold-change values and statistical significance, determined by *p*-value, were collected as previously reported [22].

### Statistical analysis

All experiments were performed at least in triplicate and the data were exhibited as the mean ± standard deviation (SD). Statistical analysis was performed using the GraphPad Prism and Statistical Package for Social Sciences 15.0 (SPSS Inc., Chicago, IL, USA). Two-sided analysis of variance (ANOVA) was used for comparison of multiple groups. Student's *t*-test or Mann–Whitney *U*-test was used for comparison of two groups Correlation between two groups was assessed by Pearson's correlation analysis. *p <* 0.05(^*^), *p <* 0.001(^**^) and *p <* 0.0001(^***^) were considered statistically significant.

## CONCLUSIONS

Our study provides the first evidence that increased TXNIP induces aggressive tumor behaviors in HCC cells by increasing cellular ROS level, motility and invasion. Future studies will not only elucidate the molecular mechanisms underlying such regulation but will also reveal whether this is a universal regulation among common cancers.

## SUPPLEMENTARY MATERIALS FIGURES AND TABLES



## Abbreviations

ANOVA: Analysis of variance
DPI: days post-injection
EMT: Epithelial mesenchymal transition
HCC: Hepatocellular carcinoma
ROS: Reactive oxygen species
TXNIP: Thioredoxin interacting protein
WB: Western blotting.
