# Supplementary material for: Thioredoxin interacting protein promotes invasion in hepatocellular carcinoma
Source: Oncotarget. 2018 Dec 7;9(96):36849–66. doi: 10.18632/oncotarget.26402 (PMC6305144; doi:10.18632/oncotarget.26402)
Supplement: Supplementary file 1 [file oncotarget-09-36849-s001.pdf]

Thioredoxin interacting protein promotes invasion in hepatocellular carcinoma

SUPPLEMENTARY MATERIALS

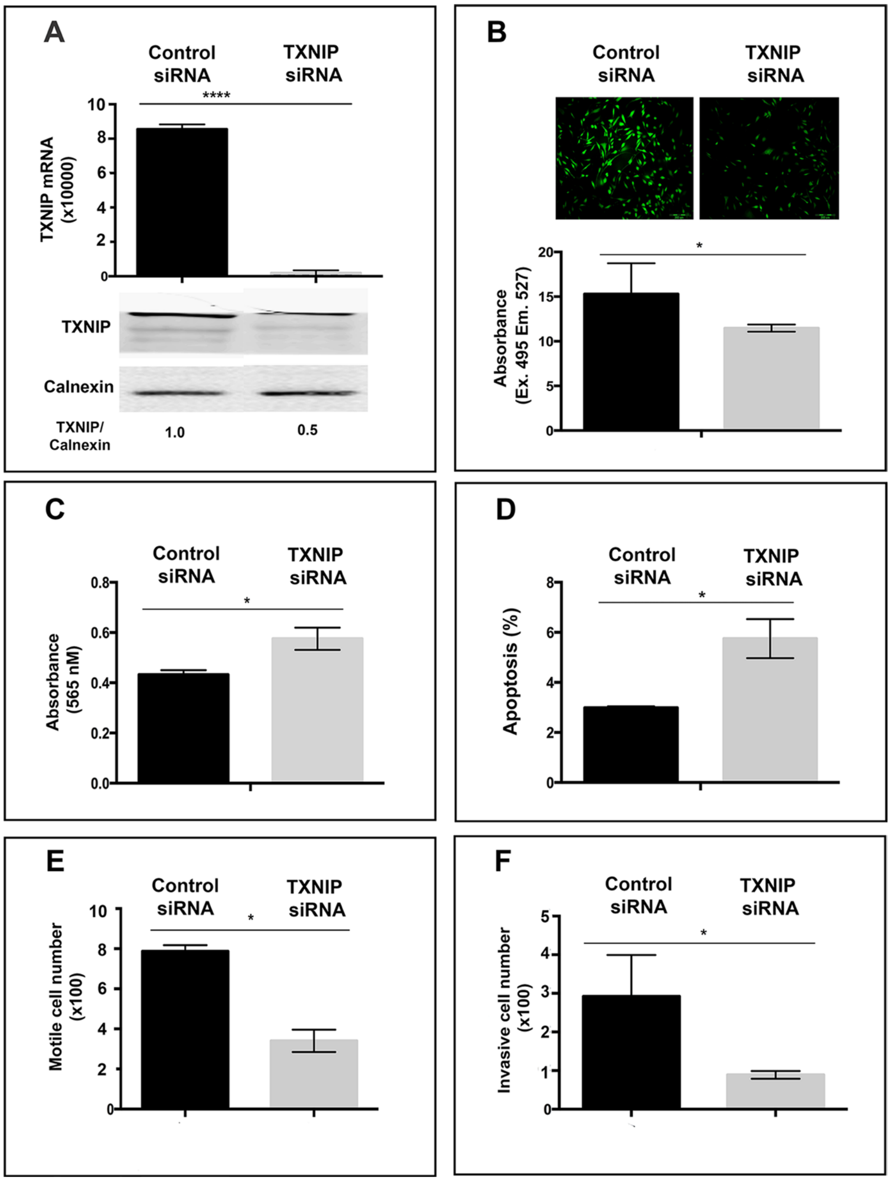

Supplementary Figure 1: Effects of TXNIP silencing on ROS levels, proliferation, apoptosis, motility and invasion in SNU-449 cells. (**A**) TXNIP mRNA and protein levels were examined by qPCR and WB, respectively in TXNIP and control siRNA treated SNU-449 cells. (**B**) The effect of TXNIP silencing on ROS levels was detected as described above. (**C**) Proliferation levels of TXNIP and control siRNA treated SNU-449 cells were detected by SRB assay. (**D**) The effect of TXNIP silencing on apoptosis was determined by Annexin V-FITC/PI double staining and quantified by flow cytometer. To determine the effects of TXNIP silencing on motility and invasion Boyden chamber motility and invasion assays were used. (**E**, **F**) The results are representative of three independent experiments done in quadruplicate. Bars represent the mean number of migrated and invaded cells per well**.** Error bars ± SD (n = 3 experiments), $^{*}p < 0.05$, $^{****}p < 0.0001$.

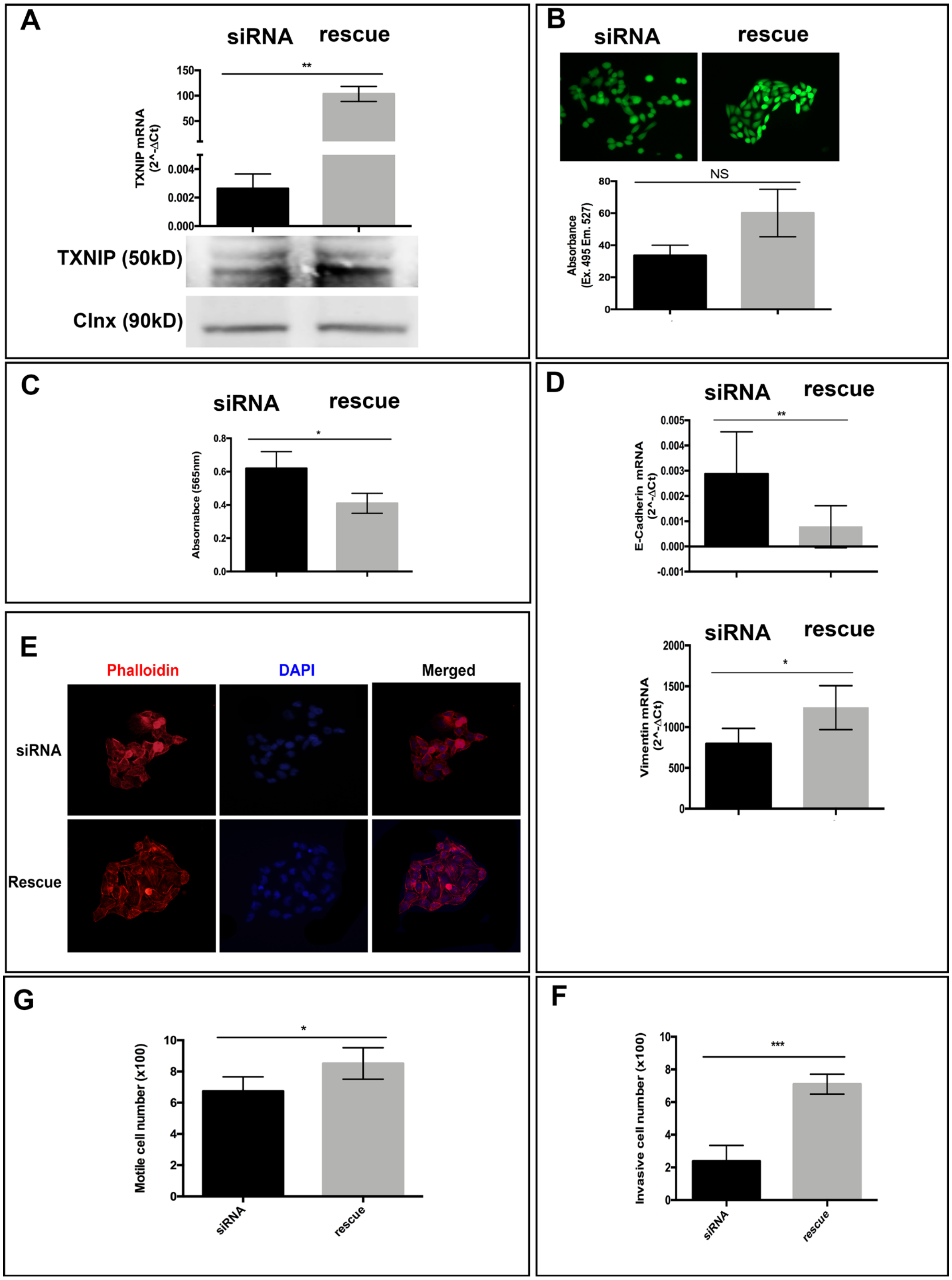

Supplementary Figure 2: The effects of TXNIP rescue on cellular ROS levels, EMT phenotype, motility and invasion of HCC cells. (**A**) SK-HEP1 cells were treated with TXNIP siRNA for 24 hours, then cells were transfected with the TXNIP over-expression and MOCK vectors, TTI-GFP-TXNIP and TTI-GFP-MOCK, respectively. Efficiency of endogenous TXNIP silencing and exogenous TXNIP over-expression was analyzed by qPCR (top) and Western blotting (bottom). (**B**) The effect of TXNIP rescue on ROS levels (**C**) proliferation (**D**, **E**) EMT (**G**) motility and (**F**) invasion was detected as described in materials methods section. The minimum value was accepted as "1" and the others were rated upon it. Error bars ± SD (n = 3 experiments). $^{*}p < 0.05$, $^{**}p < 0.01$, $^{***}p < 0.001$, $^{****}p < 0.0001$.

Supplementary Table 1: List of primer sequences used for gene expression analysis by RT-PCR

Genes	Primer sequences(5′ to 3′)*
TXNIP	F: GAGCCAGCCAACTCAAGAGA<br>R: TAGCAGACACAGGTGCCATTA
E-cadherin	F: TACACTGCCCAGGAGCCAGA<br>R: TGGCACCAGTGTCCGGATTA
Vimentin	F: CCTTGACATTGAGATTGCCACCTA<br>R: TCATCGTGATGCTGAGAAGTTTGC

*F: Forward primer. R: Reverse Primer.
